# Spectrum of germline* AIRE* mutations causing APS-1 and familial hypoparathyroidism

**DOI:** 10.1530/EJE-21-0730

**Published:** 2022-05-04

**Authors:** Treena Cranston, Hannah Boon, Mie K Olesen, Fiona J Ryan, Deborah Shears, Rosemary London, Hussam Rostom, Taha Elajnaf, Rajesh V Thakker, Fadil M Hannan

**Affiliations:** 1 Oxford Genetics Laboratories, Churchill Hospital, Oxford, UK; 2 Academic Endocrine Unit, Radcliffe Department of Medicine, University of Oxford, Oxford, UK; 3 Paediatric Endocrinology, Children's Hospital, John Radcliffe Hospital, Oxford, UK; 4 Oxford Centre for Genomic Medicine, Nuffield Orthopaedic Centre, Oxford, UK; 5 Nuffield Department of Women’s and Reproductive Health, University of Oxford, Oxford, UK; 6 National Institute for Health Research Oxford Biomedical Research Centre, Oxford, UK

## Abstract

**Objective:**

The autoimmune polyendocrine syndrome type 1 (APS-1) is an autosomal recessive disorder characterised by immune dysregulation and autoimmune endocrine gland destruction. APS-1 is caused by biallelic mutations affecting the autoimmune regulator (*AIRE*) gene on chromosome 21q22.3, which facilitates immunological self-tolerance. The objective was to investigate >300 probands with suspected APS-1 or isolated hypoparathyroidism for* AIRE* abnormalities.

**Methods:**

Probands were assessed by DNA sequence analysis. Novel variants were characterised using 3D modelling of the AIRE protein. Restriction enzyme and microsatellite analysis were used to investigate for uniparental isodisomy.

**Results:**

Biallelic *AIRE* mutations were identified in 35 probands with APS-1 and 5 probands with isolated hypoparathyroidism. These included a novel homozygous p.(His14Pro) mutation, predicted to disrupt the N-terminal caspase activation recruitment domain of the AIRE protein. Furthermore, an apparently homozygous *AIRE* mutation, p.Leu323fs, was identified in an APS-1 proband, who is the child of non-consanguineous asymptomatic parents. Microsatellite analysis revealed that the proband inherited two copies of the paternal mutant* AIRE* allele due to uniparental isodisomy. Hypoparathyroidism was the most common endocrine manifestation in *AIRE* mutation-positive probands and >45% of those harbouring *AIRE* mutations had at least two diseases out of the triad of candidiasis, hypoparathyroidism, and hypoadrenalism. In contrast, type 1 diabetes and hypothyroidism occurred more frequently in *AIRE* mutation-negative probands with suspected APS-1. Around 30% of *AIRE* mutation-negative probands with isolated hypoparathyroidism harboured mutations in other hypoparathyroid genes.

**Conclusions:**

This study of a large cohort referred for *AIRE* mutational analysis expands the spectrum of genetic abnormalities causing APS-1.

## Introduction

Autoimmune polyendocrine syndrome type 1 (APS-1; OMIM #240300), also referred to as the autoimmune polyendocrinopathy–candidiasis–ectodermal dystrophy (APECED) syndrome, is an autosomal recessive disorder characterised by immune dysregulation and multi-system autoimmune disorders, particularly affecting endocrine organs such as the parathyroid glands and adrenal cortex (1, 2). It is clinically defined by the occurrence of at least two disease components out of the triad of chronic mucocutaneous candidiasis, hypoparathyroidism, and adrenal insufficiency (1, 3). APS-1 is associated with a broad range of autoimmune diseases such as gonadal failure, type 1 diabetes, hyper- and hypo-thyroidism, autoimmune hepatitis, pernicious anemia, exocrine pancreatitis, alopecia, vitiligo, keratoconjunctivitis, pneumonitis, and tubulointerstitial nephritis, which may cause end-stage renal failure (1, 3, 4, 5). Patients with APS-1 may additionally develop ectodermal disorders such as tooth enamel hypoplasia and nail dystrophy (1, 3). APS-1 is considered to be a rare disorder with a prevalence of around 1 per 100 000 in most populations (6). However, it occurs more commonly in some geographical regions and countries such as Sardinia (1 in 14 400) and Finland (1 in 25 000), and in certain populations, for example, Iranian Jews (1 in 9000), likely due to founder effects (1, 6).

**Figure 1:**
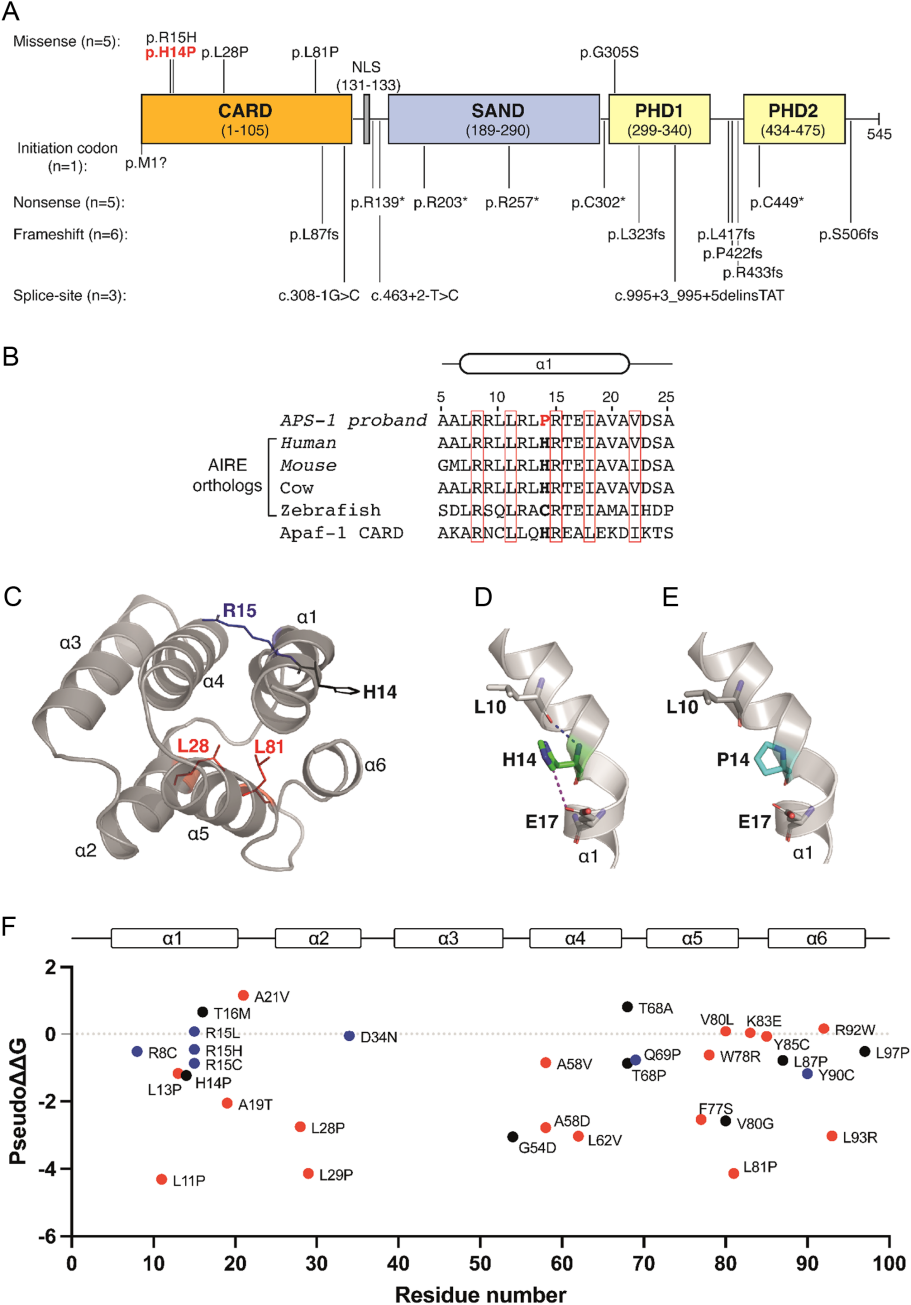
*In silico* and structural analysis of *AIRE* mutations. (A) Schematic representation of the 545-amino acid AIRE protein predicted to comprise an N-terminal caspase activation recruitment domain (CARD; residues 1–105) involved in protein multimerization (10, 20); a monopartite nuclear localization sequence (NLS; residues 131–133) (32); a SAND domain (named after proteins harbouring this domain: Sp100, AIRE, NucP1/P75, DEAF-1; residues 189–290) involved in protein–protein interactions and DNA binding (3, 18); plant homeodomain 1 (PHD1; residues 299–340), which interacts with histone H3 (19); and plant homeodomain 2 (PHD2; residues 434–475), which may interact with protein complexes promoting transcriptional elongation (33). The location of the 20 different mutations identified in 40 probands is shown. The novel p.His14Pro variant is shown in red. (B) Multiple protein sequence alignment of the N-terminal α1-helix of AIRE orthologs showing conservation of the WT His14 (bold) residue in mammals and in the homologous apoptotic protease-activating factor 1 (Apaf-1) CARD. The mutant Pro14 residue identified in an APS-1 proband (Table 1) is shown in red. Conserved or homologous residues are shown in red boxes. (C) AlphaFold 3D structure of the AIRE CARD (16). The AIRE CARD is predicted to comprise six alpha helices (α1–α6), and the location of the mutated His14 (H14), Arg15 (R15), Leu28 (L28), and Leu81 (L81) residues identified in this study are shown. (D) The WT His14 residue (green) is predicted to form interactions with Leu10 and Glu17 α1-helix residues. (E) These interactions are disrupted by the introduction of the mutant Pro14 residue (cyan). (F) Graph showing predicted effect of all AIRE CARD missense mutations on CARD domain stability (9, 21). Mutations are plotted according to residue number (*x*-axis) and the predicted stability difference score (pseudo ∆∆G) is shown on the *y*-axis. Mutations with negative pseudo ∆∆G values are predicted to impair protein stability (17). Neutral, hydrophilic, and hydrophobic residues mutated in APS-1 are shown in black, blue, and red, respectively.

APS-1 is caused by biallelic mutations of the autoimmune regulator (*AIRE*) gene located on chromosome 21q22.3. *AIRE* comprises 14 exons, which encode a 545-amino acid multidomain protein expressed predominantly in a subset of medullary thymic epithelial cells (mTECs) (3). The AIRE protein promotes central immune tolerance by increasing ectopic expression of a repertoire of tissue-specific antigens within mTECs, thereby allowing these peptides to be presented to developing T cells in the thymus, which leads to the negative selection of auto-reactive T cell clones and also induces positive selection of regulatory T cells involved in the prevention of autoimmunity (3, 6, 7). AIRE functions as a transcriptional regulator and forms multimeric nuclear protein complexes that localise to super-enhancer chromatin regions within mTECs (8). These AIRE complexes interact with topoisomerases and likely enhance mTEC gene expression by promoting transcriptional elongation at the transcription start sites of tissue-specific antigens (8). Greater than 150 different disease-causing *AIRE* mutations are reported in the Human Gene Mutation Database (HGMD), ranging from missense mutations to gross deletions encompassing part or all of the *AIRE* coding region (9). Such mutations have provided insights into AIRE structure–function and revealed protein domains critical for nuclear localisation, complex formation, and transcriptional activity (Fig. 1) (10, 11). Furthermore, individual *AIRE* mutations are associated with variable APS-1 phenotypes, even amongst affected siblings (5), thus highlighting the potential involvement of additional genetic and environmental factors. Four *AIRE* mutations have been shown to commonly occur in APS-1 patients and these are p.(Arg257Ter) in Finnish, German, Swiss, British, and Northern Italian families; p.(Arg139Ter) in Sardinian families; p.(Tyr85Cys) in Iranian Jewish families; and a 13-bp deletion in exon 8 in British, Dutch, German, and Finnish families (12, 13, 14). Here, we report the identification of biallelic *AIRE* mutations in 40 probands, which include a novel p.(His14Pro) mutation located within the N-terminal AIRE domain. In addition, we demonstrate paternal uniparental isodisomy (UPiD) of chromosome 21q as a novel cause of APS-1.

## Subjects and methods

### Subjects

Three hundred five index cases were referred to our centre in Oxford (UK) between 2005 and 2020 for constitutional analysis of the *AIRE* gene. These cases consisted of 144 probands with suspected APS-1 who had been referred for *AIRE* analysis and 161 probands with suspected familial hypoparathyroidism who either had stepwise analysis of hypoparathyroidism genes such as *AIRE, CASR, GATA3, GCM2* or were referred for analysis of a gene panel comprising *AIRE, CASR, GATA3, GCM2*, *GNA11,* and* PTH,* with* TBCE* included in this panel from 2019 onwards (Table 1 and Supplementary Tables 1, 2, see section on supplementary materials given at the end of this article). This study was approved by the University of Oxford Joint Research Office and Oxford University Hospitals NHS Foundation Trust.

**Table 1:** Clinical and DNA sequence findings in 40 probands with *AIRE* mutations.

Proband	Age (years)^a^	Clinical features^c^	Nucleotide change^d,e^	Predicted amino acid change^e^	Exon/intron
1	23	H, C, AH, V, E, P	c.1A>G(;)(1A>G)^h^	p.(Met1?)(;)(Met1?)^f^	Exon 1
2	9	H^j^	c.41A>C(;)(41A>C)^g,h,i^	p.(His14Pro)(;)(His14Pro)	Exon 1
3	14	H, C, AD, T	c.44G>A(;)(44G>A)^h^	p.(Arg15His)(;)(Arg15His)	Exon 1
4	8^b^	APECED	c.83T>C^k^	p.(Leu28Pro)^k^	Exon 1
5	9^b^	H, AD, DM	c.[242T>C];[1265delC]	p.[(Leu81Pro)];[(Pro422fs)]	Exon 2; Exon 10
6	13	C, AD, AL	c.260delT(;)(260delT)^h^	p.(Leu87fs)(;)(Leu87fs)	Exon 2
7	17	H, C	c.260delT(;)(260delT)^h^	p.(Leu87fs)(;)(Leu87fs)	Exon 2
8	11	H^j^	c.260delT(;)(260delT)^h^	p.(Leu87fs)(;)(Leu87fs)	Exon 2
9	17	H, C	c.308-1G>C(;)(308-1G>C)^l^	- ^f^	Intron 2
10	11	H, AD, PO	c.[415C>T];[967_979del13]	p.[(Arg139Ter)];[(Leu323fs)]	Exon 3, Exon 8
11	10	- ^m^	c.463+2T>C(;)(463+2T>C)^g,h,l^	- ^f^	Intron 3
12	12	H^n^	c.463+2T>C^g,l^ (;)967_979del13^h^	(-^ f^)(;) p.(Leu323fs)	Intron 3; Exon 8
13	12	H^j^	c.607C>T(;)(607C>T)^h^	p.(Arg203Ter)(;)(Arg203Ter)	Exon 5
14	10	- ^m^	c.769C>T(;)(769C>T)^h^	p.(Arg257Ter)(;)(Arg257Ter)	Exon 6
15	19	H, ND, EH, AL, TN	c.769C>T(;)967_979del13^h^	p.(Arg257Ter)(;)(Leu323fs)	Exon 6; Exon 8
16	5	H^n^	c.769C>T(;)967_979del13^h^	p.(Arg257Ter)(;)(Leu323fs)	Exon 6; Exon 8
17	17	- ^m^	c.[906T>A];[995+3_995+5delinsTAT]^g,l^	p.[Cys302Ter]; - ^f^	Exon 8; Intron 9
18	49	C	c.[913G>A]^g^;[967_979del13]	p.[(Gly305Ser)];[(Leu323fs)]	Exon 8
19	6^b^	H, AD, EH	c.967_979del13^o^	p.(Leu323fs)	Exon 8
20	4	- ^m^	c.967_979del13(;)(967_979del13)^h^	p.(Leu323fs)(;)(Leu323fs)	Exon 8
21	9	- ^m^	c.967_979del13(;)(967_979del13)^h^	p.(Leu323fs)(;)(Leu323fs)	Exon 8
22	11	H, AD	c.[967_979del13];[967_979del13]	p.[(Leu323fs)];[(Leu323fs)]	Exon 8
23	53	APECED	c.967_979del13(;)(967_979del13)^h^	p.(Leu323fs)(;)(Leu323fs)	Exon 8
24	5	- ^m^	c.967_979del13(;)(967_979del13)^h^	p.(Leu323fs)(;)(Leu323fs)	Exon 8
25	11	H^j^	c.[967_979del13];[967_979del13]	p.[(Leu323fs)];[(Leu323fs)]	Exon 8
26	39	H, C, AD	c.967_979del13(;)(967_979del13)^h^	p.(Leu323fs)(;)(Leu323fs)	Exon 8
27	2	H^n^	c.[967_979del13];[967_979del13]	p.[(Leu323fs)];[(Leu323fs)]	Exon 8
28	62	H, AD, T, PO	c.967_979del13(;)(967_979del13)^h^	p.(Leu323fs)(;)(Leu323fs)	Exon 8
29	10^b^	H, AD	c.967_979del13(;)(967_979del13)^h^	p.(Leu323fs)(;)(Leu323fs)	Exon 8
30	3	H, C	c.[967_979del13];[967_979del13]	p.[(Leu323fs)];[(Leu323fs)]	Exon 8
31	16	H^j^	c.967_979del13(;)(967_979del13)^h^	p.(Leu323fs)(;)(Leu323fs)	Exon 8
32	4^b^	H^n^	c.[967_979del13];[967_979del13]	p.[(Leu323fs)];[(Leu323fs)]	Exon 8
33	5^b^	H, AD	c.[967_979del13];[1265delC]	p.[(Leu323fs)];[(Pro422fs)]	Exon 8; Exon 10
34	9	- ^m^	c.967_979del13(;)1295_1296insA^h^	p.(Leu323fs)(;)(Arg433fs)	Exon 8; Exon 11
35	18	H, AH	c.967_979del13(;)1347C>A^h^	p.(Leu323fs)(;)(Cys449Ter)	Exon 8; Exon 11
36	14	- ^m^	c.1249dupC(;)(1249dupC)^h^	p.(Leu417fs)(;)(Leu417fs)	Exon 10
37	9	H^n^	c.[1249dupC];[1249dupC]	p.[(Leu417fs)];[(Leu417fs)]	Exon 10
38	33	- ^m^	c.1249dupC(;)(1249dupC)^h^	p.(Leu417fs)(;)(Leu417fs)	Exon 10
39	6	- ^m^	c.[1517delG];[1517delG]	p.[(Ser506fs)];[(Ser506fs)]	Exon 13
40	7	H^n^	c.[1517delG];[1517delG]	p.[(Ser506fs)];[(Ser506fs)]	Exon 13

^a^Age at the time of referral. ^b^Age at initial presentation or diagnosis. ^c^Clinical features: H, hypoparathyroidism; C, candidiasis; AH; autoimmune hepatitis; V, vitiligo; E, enteropathy; P, pancreatic insufficiency; AD, adrenal insufficiency; T, hypothyroidism; DM, type 1 diabetes; AL, alopecia; EH, enamel hypoplasia; ND, nail dystrophy; TN, tubulointerstitial nephritis; PO, premature ovarian failure; APECED, reported to have clinical features of APECED syndrome. ^d^Nucleotides are numbered according to the *AIRE* cDNA reference sequence (NM_000383.3). ^e^Nucleotide and amino acid changes have been described according to Human Genome Variation Society nomenclature guidelines (http://varnomen.hgvs.org). Use of parentheses around the amino acid change indicates that this is a prediction based on the nucleotide substitution. Where variants are confirmed to be on different alleles (*in trans*), by family studies, the alleles are described in square brackets separated by a semicolon. Absence of square brackets and use of a semicolon in parentheses indicates prediction of *in trans* variants. Use of parentheses around the second allele in the nucleotide description indicates prediction of homozygosity (family studies not available to confirm). ^f^The effect of the variant at the protein level cannot be predicted. ^g^Variant classified as likely pathogenic (class 4 variant). ^h^Homozygosity or compound heterozygosity is assumed in the proband based on their genotype and phenotype, as family studies were not possible. ^i^Novel variant. ^j^Mutation or variant identified from analysis of hypoparathyroidism genes rather than from isolated *AIRE* analysis. ^k^Compound heterozygous mutation with unbalanced translocation resulting in monosomy for *AIRE,* and a missense substitution affecting the remaining *AIRE* allele. ^l^Intronic variant predicted to affect splicing efficiency. ^m^-, not available. ^n^Clinical details incomplete. ^o^Mutation associated with uniparental isodisomy.

**Table 2:** Comparison of age and clinical features between probands with *AIRE* mutations and probands with suspected autoimmune polyendocrine syndrome type 1 (APS-1) who do not harbour an *AIRE* mutation.

Clinical features	Mutation-positive probands^a^	Mutation-negative probands^a^
*n*	30	90
Age (years)^b^	17.0 ± 14.7	23.1 ± 19.5
≥2 components of APS-1 disease triad	47%	16%*
Endocrine features		
Hypoparathyroidism	87%	32%***
Adrenal insufficiency	33%	44%
Type 1 diabetes	3%	29%**
Hypothyroidism	7%	37%**
Premature ovarian failure	7%	2%
Oral, skin, or nail features		
Candidiasis	27%	22%
Enamel hypoplasia	7%	3%
Nail dystrophy/infections	3%	3%
Alopecia	7%	6%
Vitiligo	3%	9%
Gastrointestinal features		
Autoimmune hepatitis	7%	7%
Vitamin B12 deficiency	0%	2%
Enteropathy/intestinal dysfunction	3%	1%
Pancreatic insufficiency	3%	0%
Renal features		
Tubulointerstitial nephritis	3%	0%

Differences in age were analysed using unpaired *t*-test with Welch’s correction for unequal variances. Differences in the proportions of clinical features were analysed using chi square test.

*P  < 0.05, **P  < 0.01, ***P  < 0.0001 for a comparison of mutation-positive and mutation-negative probands. ^a^Number of probands with available clinical details. ^b^Age was shown as mean ± s.d.

### Mutational analysis

Gene analysis was performed using leucocyte DNA obtained following informed consent from affected probands. Sanger sequencing of all coding exons and exon–intron boundaries was undertaken with exon-specific m13-tagged primers (IDT technologies), and using the BigDye Terminator v3.1 Cycle Sequencing Kit (ThermoFisher), and an automated detection system (ABI3730 Automated capillary sequencer; Applied Biosystems), as reported (15). Identified variants were investigated for likelihood of pathogenicity in accordance with the Association for Clinical Genomic Science best practice guidelines (https://www.acgs.uk.com/quality/best-practice-guidelines/). Sanger sequencing was undertaken in available family members following identification of pathogenic variants in probands. The population frequency of novel *AIRE* variants was assessed using the publicly accessible Genome Aggregation Database (gnomAD) (https://gnomad.broadinstitute.org/). The functional consequence of novel *AIRE* variants was predicted using Polyphen-2 (http://genetics.bwh.harvard.edu/pph2/) and the Rare Exome Variant Ensembl Learner (REVEL) (https://sites.google.com/site/revelgenomics/).

### Analysis of uniparental isodisomy

Parental investigation of the *AIRE* mutation identified in a proband with suspected UPiD was undertaken by restriction enzyme analysis, as described (14). This was further analysed using chromosome 21q microsatellite markers, which were genotyped by fluorescent PCR combined with capillary electrophoresis (ABI3730 genetic analyzer (Applied Biosystems)), as follows: the D21S2180 marker was analysed using internal marker-specific primers and GeneMapper software (Applied Biosystems), whereas the D21S1435, D21S11, D21S1444, D21S1437, D21S1411, and D21S1442 markers were analysed using the Devyser Compact QF-PCR kit (Devyser AB), and the D21S1446 and D21S1409 markers were analysed using the Elucigene QST*R-21 Euplex kit (Gen-Probe/Hologic).

### Protein sequence alignment and structural analysis of AIRE mutations

Protein sequences of AIRE orthologs were aligned using Homologene (https://www.ncbi.nlm.nih.gov/homologene). A 3D structure of the human AIRE protein (Uniprot: O43918) was obtained from the AlphaFold Protein Structure Database (https://alphafold.ebi.ac.uk/). AlphaFold is a neural network method that predicts 3D protein structures based on their amino acid sequence with near-experimental accuracy (16). The AlphaFold N-terminal AIRE CARD domain structure is predicted to be highly accurate with a predicted local-distance difference test score of >90 (16). The AIRE CARD domain structure was used to model missense *AIRE* mutations identified in this study and those also reported in HGMD (http://www.hgmd.cf.ac.uk/ac) (9). *AIRE* mutation modelling was performed using The PyMOL Molecular Graphics System (Version1.2r3pre, Schrödinger, LL Pymol). Site Directed Mutator (SDM) was used to determine the predicted effect of AIRE CARD domain mutations on protein stability (17). SDM estimates the thermal stability change between WT and mutant protein, giving a predicted stability difference score (pseudo ∆∆G) (17). A stability difference score was determined for each reported mutation in the domain.

### Statistical analysis

The analysis of clinical variables was undertaken using GraphPad Prism 9 (GraphPad). Data are presented as either mean ± s.d. or as percentages. Differences in proband age were analysed using an unpaired *t*-test with Welch’s correction for unequal variances. Differences in the proportions of clinical features were analysed using chi square test. *P* value <0.05 was considered statistically significant for all analyses.

## Results

### Overview of probands harbouring AIRE variants

DNA sequence analysis of the 14 exons and exon–intron boundaries of the *AIRE* gene in 305 index cases resulted in the detection of germline mutations in 40 unrelated probands (Table 1). Thirty-two probands (80%) were children with a mean ± s.d. age of 9.6 ± 4.2 years, and eight probands were adults with a mean ± s.d. age of 37.0 ± 16.6 years (Table 1). Thirty-five probands comprised children and adults with suspected APS-1, who had been referred for *AIRE* testing, and five probands were children who were referred for analysis of hypoparathyroidism genes (Table 1). Hypoparathyroidism was the most common endocrine disorder in this patient cohort and was diagnosed in 26 out of 30 probands (87%) with available clinical details (Table 1). Other endocrine disorders affecting the probands in this study included adrenal insufficiency, hypothyroidism, type 1 diabetes, and premature ovarian failure (Table 1). Thirteen probands had non-endocrine autoimmune diseases comprising candidiasis, autoimmune hepatitis, vitiligo, enteropathy, pancreatic insufficiency, alopecia, enamel hypoplasia, nail dystrophy, and tubulointerstitial nephritis (Table 1).

The clinical features of probands with *AIRE* mutations were compared to probands with suspected APS-1, who did not harbour an *AIRE* mutation (Table 2 and Supplementary Table 1). This revealed that >45% of probands with *AIRE* mutations had at least two diseases out of the triad of chronic mucocutaneous candidiasis, hypoparathyroidism, and adrenal insufficiency (Table 2). Whereas, significantly fewer *AIRE* mutation-negative probands with suspected APS-1 (~16%, *P*  < 0.01) had developed two out of three components of the classic disease triad (Table 2). Hypoparathyroidism occurred significantly more frequently (*P*  < 0.0001) in probands with *AIRE* mutations, whereas type 1 diabetes and hypothyroidism occurred significantly more frequently (*P*  < 0.01) in *AIRE* mutation-negative probands (Table 2). An analysis of probands with suspected familial hypoparathyroidism showed that the five probands harbouring *AIRE* mutations all had apparently isolated hypoparathyroidism and were significantly younger than the *AIRE* mutation-negative probands (mean ± s.d. age of 11.8 ± 2.6 vs 20.6 ± 20.3 years, *P*  < 0.001) (Table 1 and Supplementary Table 2). Greater than 90% of *AIRE* mutation-negative probands with suspected familial hypoparathyroidism had apparently isolated hypoparathyroidism, and around 30% of these probands were found to have an abnormality affecting either the *CASR, GATA3, GCM2*, *GNA11* genes or the 22q11.2 chromosomal region (Supplementary Table 2).

### Analysis of AIRE mutations

Twenty different *AIRE* mutations were detected in the 40 probands (Fig. 1A and Table 1). All mutations occurred in the compound heterozygous or homozygous state and comprised six frameshift, three splice-site, five missense, five nonsense, and one initiation codon mutation that potentially alters protein translation (Fig. 1A and Table 1). The five missense mutations affect key AIRE domains (3, 18). Thus, one reported missense mutation, p.(Gly305Ser), is located in the first plant homeodomain (PHD1) zinc finger, which is involved in histone interactions (19), whilst four missense mutations are located in the N-terminal caspase activation recruitment domain (CARD) of AIRE, which mediates protein multimerisation (Fig. 1A) (10). These four AIRE CARD mutations comprised a novel homozygous missense substitution, c.41A>C; p.(His14Pro), identified in a child with hypoparathyroidism (proband 2, Table 1) and the previously reported p.(Arg15His), p.(Leu28Pro), and p.(Leu81Pro) missense mutations (Fig. 1A and Table 1) (20, 21, 22). Bioinformatic analyses using Polyphen-2 and REVEL predicted the novel p.(His14Pro) variant to be probably damaging (Polyphen-2 score 0.97, REVEL score 0.69). Furthermore, the p.(His14Pro) variant was shown to affect a residue evolutionarily conserved in mammals, but not in zebrafish (Fig. 1B), and this variant was not detected in the gnomAD database. To determine the mechanism by which p.(His14Pro) and the other reported missense CARD mutations identified in this study may affect AIRE function, we used a neural network-derived 3D structure of the human AIRE protein, which is available from the AlphaFold Protein Structure Database (https://alphafold.ebi.ac.uk/) (16). The AlphaFold structure was utilised as no crystal or cryo-EM structures of the AIRE CARD have been reported. The N-terminal AIRE CARD domain was shown to comprise six alpha helices clustered around a core region of highly conserved hydrophobic residues (Fig. 1C) (20). Structural analysis of the four AIRE CARD missense mutations identified in this study (Fig. 1A and Table 1) showed that the novel p.(His14Pro) variant and reported p.(Arg15His) mutation affect externally facing residues located within the α1 helix of the CARD domain (Fig. 1C), and which are predicted to comprise part of the interface involved in forming multimeric protein complexes (10). Thus, these mutations may potentially disrupt this interface and impair AIRE protein multimerisation (10). The novel p.(His14Pro) variant may be predicted to disrupt or kink the α1 helix, and 3D modelling additionally showed this variant to impair interactions with neighbouring α1 helix residues (Fig. 1D and E). Thus, these *in silico* analyses of the p.(His14Pro) *AIRE* variant indicated that this was likely pathogenic. In contrast, the reported p.(Leu28Pro) and p.(Leu81Pro) mutations (Fig. 1A and Table 1) were shown to affect hydrophobic residues located within the core of this domain (Fig. 1C) and may potentially destabilise the CARD structure and/or cause protein misfolding (20). An analysis of all 31 reported missense AIRE CARD mutations (9, 21), together with the novel p.(His14Pro) variant, demonstrated that >50% of these mutations affect hydrophobic residues (Fig. 1F). Moreover, the majority (>90%) of mutations affecting hydrophobic residues were predicted to impair stability of the CARD domain (Fig. 1F).

### Uniparental isodisomy causing APS-1

**Figure 2:**
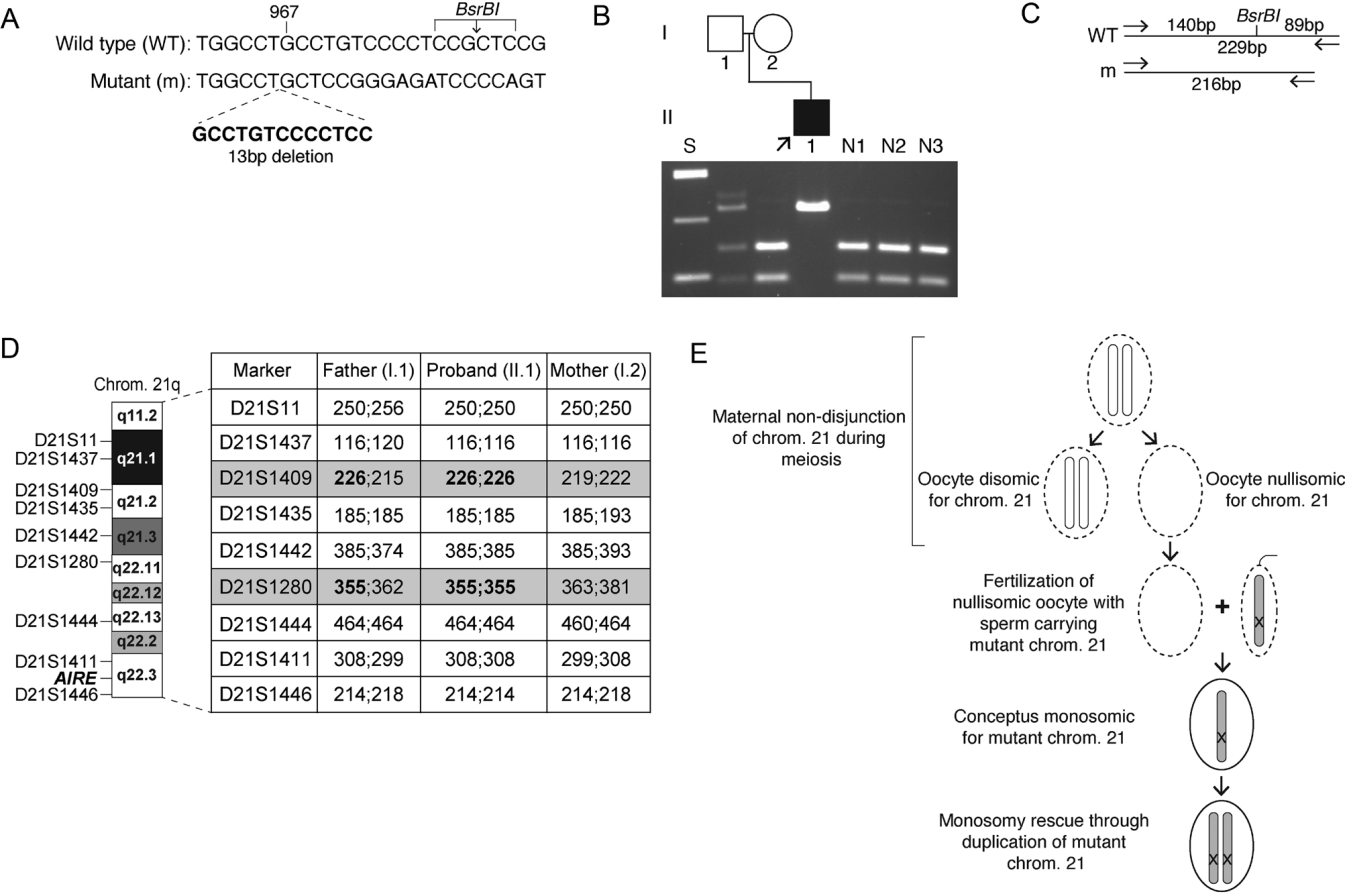
Identification of 21q uniparental isodisomy. (A) DNA sequence analysis of the *AIRE* gene in proband 19 (Table 1) revealed a 13-bp deletion (c.967_979del13, p.Leu323fs), which is predicted to result in the loss of a *BsrBI* restriction enzyme site. (B) PCR and *BsrBI* digestion confirmed that this proband (individual II.1, arrow) is homozygous for the p.Leu323fs mutation, whereas the father (individual I.1) is heterozygous for the p.Leu323fs mutation and the mother (individual I.2) is unaffected. (C) Restriction enzyme map showing that *BsrBI* digestion would result in two products of 140 bp and 89 bp from the 229-bp WT sequence but would not affect the 216-bp mutant (m) sequence, as reported (14). (D) Microsatellite analysis of chromosome 21q in the proband and parents. An analysis of nine markers across 21q21.1-21q22.3 (http://genome.ucsc.edu) showed the proband to be homozygous at all loci tested. Two markers (D21S1409 & D21S1280 (in bold and shaded grey)) were informative and demonstrated that there was no maternally derived allele. These results are consistent with paternal uniparental isodisomy (UPiD). (E) Schematic representation of the potential mechanism for paternal UPiD of the *AIRE* mutation in the proband, which may have been caused by generation of a nullisomic oocyte during meiosis and the rescue of the monosomic conceptus by duplication of the paternally derived chromosome 21 (shaded grey), which is carrying the mutant *AIRE* allele (represented by an ‘X’). Germ cells/gametes are represented by dashed ellipses, and the conceptus is represented by a solid ellipse.

The most frequent *AIRE* mutation identified in this study was a 13-bp deletional frameshift affecting exon 8 (c.967_979del13; p.Leu323fs), which is reported to commonly cause APS-1 in the British population (14). This was identified as a compound heterozygous or homozygous mutation in 22 out of 40 probands (55%) including in an individual (proband 19, Table 1; individual II.1, Fig. 2A and B), who was diagnosed with hypoparathyroidism, dental enamel hypoplasia, awnd adrenal insufficiency. The proband is the child of non-consanguineous asymptomatic parents (Fig. 2B). At conception of the proband, the mother (individual I.2, Fig. 2B) and father (individual I.1, Fig. 2B) were aged >40 years. DNA sequencing and restriction enzyme analysis of the proband showed apparent homozygosity for the *AIRE* p.Leu323fs mutation (Fig. 2B and C). However, parental analysis of the *AIRE* gene revealed the paternal DNA to be heterozygous for the p.Leu323fs mutation, whilst this mutation was absent in the maternal DNA (Fig. 2B). To establish the mode of inheritance of the p.Leu323fs *AIRE* mutation in this family, microsatellite analysis was undertaken using nine markers located across chromosome 21q. This revealed that the proband was homozygous for all loci tested, and a lack of maternal contribution was demonstrated for two fully informative markers located at chromosome 21q21.2 and 21q22.11 (Fig. 2D). Moreover, karyotype analysis of the proband and parents did not reveal any chromosome 21q deletions (data not shown). Thus, these findings are consistent with the proband having inherited two copies of the paternal mutant *AIRE* allele due to UPiD. Family studies undertaken in 12 other probands who appeared homozygous for *AIRE* mutations and had available family members (Table 1) did not reveal any further cases of uniparental disomy (UPD) or UPiD.

## Discussion

Our results, which have identified *AIRE* mutations in 40 probands, further expand the spectrum of germline abnormalities causing APS-1. Hypoparathyroidism is the most common endocrine disorder in this cohort and affected >80% of probands (Table 1). This is consistent with the reported prevalences from Finnish and American APS-1 patient cohorts, which showed that ≥85% of patients develop hypoparathyroidism by the age of 30 years (1, 5). Moreover, five probands with apparent isolated hypoparathyroidism, and who were referred for hypoparathyroidism gene panel analysis, were found to harbour homozygous *AIRE* mutations (Table 1). Isolated hypoparathyroidism is occasionally reported in individuals harbouring biallelic *AIRE* mutations, including in adult patients who have had long-term clinical follow-up (23). However, the probands with isolated hypoparathyroidism in the present study are all children (mean age 11.8 years) (Table 1). Given that autoimmune parathyroid gland destruction represents an early manifestation of APS-1 with a peak incidence occurring at around 5 years of age (5), it remains to be established whether these probands will subsequently develop additional features of APS-1. Thus, ongoing follow-up of these hypoparathyroid probands is warranted for assessment of other endocrine and non-endocrine manifestations of APS-1. It is notable that around 30% of *AIRE* mutation-negative probands with suspected familial hypoparathyroidism harboured mutations in other genes known to cause hypoparathyroidism. This highlights the importance of investigating these probands using a panel of hypoparathyroid genes such as *CASR, GATA3, GCM2*, *AIRE, GNA11, PTH,* and* TBCE.*

Around 85% of *AIRE* mutation-positive probands were shown to harbour biallelic frameshift, nonsense, or splice-site mutations predicted to truncate the AIRE protein or lead to nonsense-mediated decay (Table 1). Five probands harboured homozygous or compound heterozygous missense *AIRE* mutations. The missense mutations were found to cluster in the predicted N-terminal CARD domain, which represents the most highly conserved region of the AIRE protein (Fig. 1A) (20). The CARD domain is a member of the death domain superfamily, which facilitates the formation of multimeric protein assemblies involved in apoptosis and inflammatory signalling (10, 24). The CARD domain has been shown to mediate protein–protein interactions, thereby resulting in the AIRE protein forming homo-multimeric filaments situated within nuclear foci (10). Such filaments are critical for AIRE function, and missense mutations located at putative interfaces between AIRE filaments have been reported to abrogate filament formation and AIRE transcriptional activity (10). Consistent with this, our structural analysis showed the novel p.(His14Pro) variant and reported p.(Arg15His) mutation to affect residues located at the surface of the AIRE CARD domain (Fig. 1C), and therefore, these mutations are predicted to disrupt AIRE–AIRE protein interactions and affect interactions with other proteins involved in transcription and mRNA processing (10). It is notable that none of the *AIRE* mutations detected in this study were mono-allelic. Such mono-allelic mutations have been shown to comprise missense substitutions that exert dominant-negative effects and cause a milder APS-1 phenotype with incomplete penetrance (11). These dominant-negative *AIRE* mutations cluster in the PHD1 zinc finger domain and impair transcription–transactivation activity of AIRE (11). We identified one reported dominant-negative *AIRE* mutation, p.Gly305Ser (11), which is located in the PHD1 domain (Fig. 1A). However, this occurred as a part of a compound heterozygous mutation (proband 18, Table 1).

Molecular genetic analyses identified UPiD of chromosome 21q as a novel cause of APS-1 in one proband (Fig. 2). UPD refers to the inheritance of two homologous chromosomes or chromosomal segments from one parent and is estimated to occur in around 1 in 2000 live births (25). It is classified as UPiD when two identical copies of a single parental homolog are inherited. Paternal UPiD was demonstrated for the affected proband in this study, as the individual was found to be homozygous for microsatellite markers present in only one paternal chromosome 21q region (Fig. 2D). UPiD may affect the whole chromosome or be confined to a chromosomal segment (26). However, it was not possible to identify an informative marker telomeric to *AIRE* as this gene is located at the distal portion of 21q (Fig. 2D), and therefore, it is uncertain if the entirety of 21q is included in the UPiD. Although the *AIRE* and microsatellite analyses, along with the normal cytogenetic karyotype, are suggestive of this, and it would be in keeping with the whole chromosome non-disjunction events commonly reported to cause trisomy 21 and Down’s syndrome (27). It is notable that the proband had no clinical features which were not attributable to homozygosity for the detected *AIRE* mutation, and therefore, there is no evidence of any further pathogenic consequence of the UPiD. UPD and UPiD do not always cause disease as affected individuals have a normal gene dosage (28), and consistent with this, paternal UPiD has been reported for chromosome 21 in the absence of any abnormal phenotypes (29). However, UPD/UPiD can clinically manifest either by disrupting genetic imprinting or as with this case unmask autosomal recessively inherited disorders if one of the parents is a heterozygous carrier and the single mutant allele segregates through UPiD in the affected offspring as a homozygous mutant allele (30). In proband 19 (Fig. 2 and Table 1), this may potentially have arisen from meiotic non-disjunction of maternal chromosome 21 (Fig. 2E). This is characterised by failure of the two homologs of the chromosome 21 pair to separate into two daughter cells during germ cell meiosis (31). Thus, meiotic non-disjunction affecting maternal gametes may have caused some oocytes to lack chromosome 21 (Fig. 2E). Fertilisation of such a nullisomic oocyte with a sperm haploid for chromosome 21 and carrying a mutant *AIRE* allele (‘mutant’ chromosome 21) will result in a zygote being monosomic for the paternally derived mutant chromosome 21 (Fig. 2E). Subsequent ‘rescue’ of the monosomic zygote occurs through chromosomal duplication during mitosis (Fig. 2E), thereby leading to paternal UPiD with homozygosity for the mutant *AIRE* allele (29, 31). It should be noted that other meiotic and mitotic events can also cause UPiD of chromosome 21 (29), and although the underlying cause remains to be elucidated, advanced maternal age is associated with an increased risk of meiotic non-disjunction and may have contributed to the UPiD in this proband. The identification of UPiD as a novel cause of APS-1 has implications for genetic counselling and family management, as autosomal recessive disorders are usually associated with a recurrence risk of 25% for each pregnancy when both parents are heterozygous mutation carriers. In contrast, the risk of recurrence is likely to be low or negligible when APS-1 is caused by UPD/UPiD (28).

In conclusion, our characterisation of a large cohort referred for *AIRE* mutational analysis expands the spectrum of genetic abnormalities causing APS-1. This has also revealed differences in the clinical presentation of *AIRE* mutation-positive and mutation-negative probands with suspected APS-1. Furthermore, the finding of APS-1 being caused by UPiD rather than through an autosomal recessive mode of inheritance highlights the importance of confirming biparental inheritance in apparently homozygous APS-1 patients in order to exclude UPD/UPiD.

## Supplementary materials

Supplementary Material

## Declaration of interest

The authors declare that there is no conflict of interest that could be perceived as prejudicing the impartiality of the research reported.

## Funding

This work was supported by a Wellcome Trust Investigator Award (106995/Z/15/Z) (R V T), National Institute for Health Research (NIHR) Senior Investigator Award (NF-SI-0514-10091) (R V T), and NIHR Oxford Biomedical Research Centre Programme Grant (R V T).

## Author contribution statement

R V Thakker and F M Hannan contributed equally to this work.
