# Supplementary material for: Spectrum of germline AIRE mutations causing APS-1 and familial hypoparathyroidism
Source: Eur J Endocrinol. 2022 May 4;187(1):111–22. doi: 10.1530/EJE-21-0730 (PMC9175554; doi:10.1530/EJE-21-0730)
Supplement: Supplementary Material [file supplementary_material.pdf]

Supplementary Data

Supplementary Table 1 Clinical and DNA sequence findings in 109 *AIRE* mutation-negative probands with suspected autoimmune polyendocrine syndrome type 1 (APS-1).

Proband	Age (years)[a]	Clinical features[c]	Gene abnormality identified
41	47	H	No
42	31	H	No
43	43	C	No
44	51	AH, V	No
45	12[b]	H, DM	*GATA3* mutation
46	46	C, AD, PO	No
47	35[b]	H, C	No
48	2	AD	No
49	19	C	No
50	9[b]	U, CH, UC, PN	No
51	17	AD, R, T, AL	No
52	4	H, DM	No
53	16	DM, AL	No
54	12[b]	H, C	No
55	14[b]	T	No
56	3	-[d]	No
57	32	C, U	No
58	14	H	No
59	3[b]	H, EH	No
60	11	H	No
61	30	C, T, I	No
62	39	-[d]	No
63	53	H, T	No
64	37	-[d]	No
65	4	-[d]	No
66	3	-[d]	No
67	12	AD, DM, CE	No
68	16	H, ND	No
69	35	AD, T	No
70	1[b]	AD	No
71	44	-[d]	No
72	6	-[d]	No
73	40[b]	AD, T, DM	No
74	28[b]	AD, DM	No
75	14[b]	H, AD	No
76	18	C, T, DM	No
77	16	-[d]	No
78	16	T, DM	No
79	47	U, T, PA	No
80	6	-[d]	No
81	9	V, AL	No
82	9	AD, DM, PN	No
83	14	AD	No
84	12	T, CE	No
85	13	HS	No
86	<18[b]	AD, T, CE	No
87	59	C, AD, T	No
88	64	C, AD, T, DM, V	No
89	2[b]	DM, T	No
90	70	-[d]	No

91	2	C, AD	No
92	10[b]	C	No
93	29	- [d]	No
94	16	- [d]	No
95	64	H	No
96	41	H, AD, V, T	No
97	3	AH, V	No
98	11	AD, DM	No
99	17	AD, DM, T	No
100	2[b]	AH	No
101	30	- [d]	No
102	6	C, AD	No
103	66	H, AD	No
104	35	AD, T	No
105	16	- [d]	No
106	16	AD	No
107	56	AH, PA	No
108	16	DM	No
109	14	C, DM, T, CE	No
110	7	DM	No
111	15	C, AD, EH	No
112	57	C, AD, DM	No
113	20	AD, DM	No
114	30	Carrier testing	No
115	11[b]	AH, DM	No
116	26	H, AD	No
117	66	AD, V	No
118	13	H	No
119	3[b]	V, T	No
120	14	C	No
121	31	- [d]	No
122	14	C	No
123	62	AD, T, EH	No
124	47	C, T, PO, PA	No
125	22	- [d]	No
126	1[b]	T	No
127	1[b]	C, AL	No
128	50	AD, DM, T	No
129	5	C, T, AH	No
130	52	DM, T, V, AL	No
131	9[b]	AD	No
132	15	AD, T	No
133	38	- [d]	No
134	5[b]	DM, CE, T	No
135	4	AD	No
136	11	AD, T	No
137	40	DM, U	No
138	48	T	No
139	13	AD	No
140	54	H, T, CE	No
141	13	AD	No
142	12	AD, T	No
143	28	- [d]	No
144	6[b]	AD, DM	No
145	4	- [d]	No
146	55	AD, DM, T	No
147	9	AD, T	No
148	15	AD, DM	No

149	<1[b]	H, C	*CASR* mutation

[a]Age at the time of referral.
[b]Age at initial presentation or diagnosis.
[c]Clinical features: H, hypoparathyroidism; C, candidiasis; CE, coeliac disease; AH; autoimmune hepatitis; V, vitiligo; AD, adrenal insufficiency; T, hypothyroidism; DM, type 1 diabetes; AL, alopecia; EH, enamel hypoplasia; ND, nail dystrophy; TN, tubulointerstitial nephritis; PO, premature ovarian failure; CH, cholangitis; Hepatic steatosis; I, intestinal dysfunction; PN, paronychia; HS, PA, pernicious anaemia/vitamin B12 deficiency; R, renal impairment; U, unspecified autoimmune conditions; UC, ulcerative colitis.
[d]Clinical details unavailable.

Supplementary Table 2 Clinical and DNA sequence findings in 156 *AIRE* mutation-negative probands with suspected familial hypoparathyroidism.

Proband	Age (years)[a]	Clinical features[c]	Gene abnormality identified
150	3	H, Dys	No
151	9[b]	H	*CASR* mutation
152	4	H	*GCM2* mutation
153	14[b]	H	No
154	14[b]	H	No
155	<1	H	No
156	23	H	No
157	15	H, RC	No
158	<1[b]	H	No
159	<1[b]	H	No
160	3[b]	H	No
161	<1[b]	H	No
162	30	PHP	No
163	16[b]	H	No
164	11	H	No
165	14[b]	H	No
166	41	H	No
167	66	H, T	No
168	34	H	No
169	25	H	No
170	35	H	No
171	21	H	No
172	56	H	No
173	12	H	No
174	<1[b]	H	No
175	51	H	*CASR* mutation
176	68	H	No
177	<1[b]	H	No
178	<1[b]	H	*CASR* mutation
179	5	H, S	*GNA11* mutation
180	39	H	No
181	79	H	No
182	3	H	No
183	10[b]	H, RC, S	No
184	<1[b]	H	No
185	20	H	No
186	31	H	*CASR* mutation
187	12	H	No
188	39	H	No
189	14	H, D	No
190	20	H	No
191	39	H, T	*GNA11* mutation
192	<1[b]	H	*CASR* mutation
193	1	H	No
194	<1[b]	H	No
195	2[b]	H	*GNA11* mutation
196	13[b]	H	No
197	42[b]	H	No
198	12	H	No
199	2	H	*CASR* mutation
200	<1[b]	H	*GCM2* mutation

201	47	H	*CASR* mutation
202	12[b]	H	No
203	65	H	*CASR* mutation
204	3	H	No
205	13[b]	H	No
206	64	H	No
207	15	H	No
208	64	H	No
209	<18[b]	H	*GCM2* mutation
210	<10[b]	H	No
211	40	H, S	No
212	<1[b]	H, Ca	No
213	24	H	*GCM2* mutation
214	27	PHP	No
215	<1[b]	H	No
216	<1[b]	H	*CASR* mutation
217	<1[b]	H	*PTH* mutation
218	2	H	*GATA3* mutation
219	47	H	*CASR* mutation
220	47	H	*GNA11* mutation
221	14[b]	H	*GNA11* mutation
222	3	H	No
223	8	H	No
224	2	H	No
225	21[b]	H	No
226	9	H	No
227	26	H	No
228	36	H	No
229	<1[b]	H, HP	No
230	31[b]	H	*GCM2* mutation
231	27	H	*CASR* mutation
232	32	H	*CASR* mutation
233	48	H	No
234	8	H, D	*GATA3* mutation
235	47	H	No
236	20[b]	H	No
237	<1[b]	H	*CASR* mutation
238	39	H	*CASR* mutation
239	62	H	No
240	19	H	*CASR* mutation
241	23[b]	H	No
242	26	H	No
243	12[b]	H	No
244	17	H	*CASR* mutation
245	6	H	*GATA3* mutation
246	29[b]	H, D	*GATA3* mutation
247	42	H	No
248	78[b]	H	No
249	23[b]	H, EH	*CASR* mutation
250	11	H	No
251	36	H	No
252	11	H	No
253	23[b]	H	No
254	3	H	No
255	22[b]	H	No
256	8	H	No
257	89	H	No
258	10	H	*GCM2* mutation

259	<1[b]	H	*TBCE* mutation
260	24	H	No
261	17[b]	H	No
262	<1[b]	H	No
263	<1[b]	H	No
264	14	H	No
265	17	H	No
266	10[b]	H	No
267	8	H, D	No
268	10	H	No
269	15[b]	H	No
270	12	H	No
271	46	H	No
272	5[b]	H	*PTH* mutation
273	33	H	*CASR* mutation
274	39	H	*CASR* mutation
275	<1[b]	H	No
276	<1[b]	H	22q11.2 deletion
277	25[b]	H	No
278	<1[b]	H	No
279	56	H, D, S	No
280	<1[b]	H	No
281	<1[b]	H	No
282	<1[b]	H	No
283	<1[b]	H	No
284	<1[b]	H	*GCM2* mutation
285	<1[b]	H, D	*GATA3* mutation
286	54	H	No
287	33	H	No
288	42	H	No
289	27	H	*GNA11* mutation
290	16[b]	H	No
291	<1[b]	H	No
292	44	H	No
293	10	H	No
294	6[b]	H	*GCM2* mutation
295	73	H	*CASR* mutation
296	32	H	No
297	53	H	*CASR* mutation
298	9[b]	H	No
299	<1[b]	H	No
300	<1[b]	H	No
301	19	H, D, R	*GATA3* mutation
302	52	H	No
303	14	H, R	*GATA3* mutation
304	<1[b]	H	No
305	1	H	*GCM2* mutation

[a]Age at the time of referral.
[b]Age at initial presentation or diagnosis.
[c]Clinical features: H, hypoparathyroidism; C, candidiasis; Ca, cardiac structural defects; D, deafness, Dys, dysmorphism; EH, enamel hypoplasia; HP, hypopituitarism; PHP, pseudohypoparathyroidism; R, renal impairment; RC, renal cysts; S, short stature; T, hypothyroidism.
